# Understanding the use of co-design methods for research involving older adults living with HIV: A scoping review protocol

**DOI:** 10.1371/journal.pone.0303580

**Published:** 2024-05-30

**Authors:** Paige Brown, Hardeep Singh, Esther Su, Luxey Sirisegaram, Sarah E. P. Munce, Andrew D. Eaton, Alice Zhabokritsky, Stuart McKinlay, Kristina M. Kokorelias

**Affiliations:** 1 Division of Geriatric Medicine, Department of Medicine, Sinai Health System and University Health Network, Toronto, ON, Canada; 2 Undergraduate Medical Education, Temerty Faculty of Medicine, University of Toronto, Toronto, ON, Canada; 3 Department of Occupational Science and Occupational Therapy, Temerty Faculty of Medicine, University of Toronto, Toronto, ON, Canada; 4 KITE, Toronto Rehabilitation Institute, University Health Network, Toronto, ON, Canada; 5 Rehabilitation Sciences Institute, Temerty Faculty of Medicine, University of Toronto, Toronto, ON, Canada; 6 Institute of Health Policy, Management and Evaluation, University of Toronto, Toronto, ON, Canada; 7 Faculty of Social Work–Saskatoon Campus, University of Regina, Regina, SK, Canada; 8 Factor-Inwentash Faculty of Social Work, University of Toronto, Toronto, ON, Canada; 9 Infectious Diseases, Department of Medicine, University Health Network, Toronto, ON, Canada; 10 Division of Infectious Diseases, Department of Medicine, University of Toronto, Toronto, ON, Canada; 11 CIHR Canadian HIV Trials Network, Vancouver, BC, Canada; Stellenbosch University, SOUTH AFRICA

## Abstract

There is a growing population of adults aged 50 years or older living with HIV, facing unique challenges in care due to age, minority status, and stigma. Co-design methodologies, aligning with patient-centered care, have potential for informing interventions addressing the complex needs of older adults with HIV. Despite challenges, co-design has shown promise in empowering older individuals to actively participate in shaping their care experiences. The scoping review outlined here aims to identify gaps in existing co-design work with this population, emphasizing the importance of inclusivity based on PROGRESS-Plus characteristics for future patient-oriented research. This scoping review protocol is informed by the Joanna Briggs Institute Manual to explore co-design methods in geriatric HIV care literature. The methodology encompasses six stages: 1) developing research questions, 2) creating a search strategy, 3) screening and selecting evidence, 4) data extraction, 5) data analysis using content analysis, and 6) consultation with key stakeholders, including community partners and individuals with lived experience. The review will involve a comprehensive literature search, including peer-reviewed databases and gray literature, to identify relevant studies conducted in the past 20 years. The inclusive criteria focus on empirical data related to co-design methods in HIV care for individuals aged 50 or older, aiming to inform future research and co-design studies in geriatric HIV care. The study will be limited by the exclusion of papers not published or translated to English. Additionally, the varied terminology used to describe co-design across different research may result in the exclusion of articles using alternative terms. The consultation with key stakeholders will be crucial for translating insights into meaningful co-design solutions for virtual HIV care, aiming to provide a comprehensive synthesis that informs evidence-based strategies and addresses disparities in geriatric HIV care.

## Introduction

The population of adults aged 50 years or older living with human immunodeficiency virus (HIV), a chronic infection, is continuing to grow as advancements in HIV care, including antiretroviral therapy (ART), have become available. In 2021, approximately 63,000 individuals in Canada and 38.3 million individuals worldwide were living with HIV [1, 2]. It is further estimated that 50% of individuals living with HIV are aged 50 years or older in Canada. Globally, other countries are identifying similar trends; for example, in the Netherlands, 73% of individuals living with HIV will be aged 50 years or older by 2030 [3, 4]. Further complicating the care provided to older adults living with HIV are the barriers to care faced by this population group. Older adults living with HIV are more likely to be from underserved populations, exemplified by data showing that 49% of older adults with HIV belong to minority groups. These findings may present challenges to receiving culturally appropriate care [5]. Moreover, this patient group may face discrimination and stigma due to their HIV-positive status, leading to higher levels of loneliness and smaller social support networks. Collectively, these barriers to care, in addition to ageism, may contribute to worse health outcomes and decreased quality of life [4, 6, 7].

Co-design approaches have become increasingly prominent in healthcare research as they align with the emerging priority of patient-centred care. Furthermore, co-design approaches may also hold benefits in research design methods for work with older adults with HIV. Co-design and related terms, including co-production or co-creation, refer to research approaches to design new care interventions or care improvements that engage end-users, health care staff, and related advocacy groups in an equal collaboration [8]. Originating from community-based participatory design, co-design allows for immersive, reflective feedback from the user throughout the entirety of a project, from conceptualization to consultation [9]. The aim of engaging end-users in research is to ensure that research results can be meaningful, relevant, and useful to the population they intend to benefit [10, 11].

Although challenges exist for co-design methods involving older people, it has been noted that older people are interested in participating in the design of their own care [12]. One study used co-design in the creation of cultural mental health interventions for older adults living in Hong Kong, finding that there was value in an active participatory process and that the approach was perceived by participants to be “empowering” [13]. Historically, in HIV research, community-based participatory research methods have been conducted to address HIV prevention, care, and treatment efforts. Engagement with individuals living with HIV as partners in research design, rather than the population of interest, [14, 15] and the development of co-design and community-based research methods in response to the HIV epidemic, has been fundamental to community engagement [16]. In the context of HIV care, co-design has been used in several studies to engage relevant stakeholders, including individuals with HIV and patient partners [17–19]. Consequently, while traditional approaches may not fully address the complex needs of older adults living with HIV, co-design methods have the potential to tailor interventions to meet this group’s unique needs and empower participants to have a voice in shaping their own care experiences. The inclusion of marginalized or underrepresented perspectives will be particularly important for tailoring culturally responsive care models that meet the specific needs of older adults living with HIV. Co-designed interventions have the potential to enhance patient-provider communication and overall healthcare experiences in a culturally responsive manner. This scoping review aims to understand the gaps existing in co-design work with older adults with HIV, as no known synthesis of information exists at present. Specifically, understanding what groups of individuals may be excluded from co-design methods in relation to PROGRESS-Plus characteristics (eg, place of residence, race/ethnicity/culture/language, occupation, gender/sex, religion, education, socioeconomic status, social capital) [20] will inform future co-design research with older adults with HIV to emphasize patient-oriented research [21].

## Methods

To better understand co-design methods used in the context of research methods and interventions for older adults living with HIV, we plan to complete a scoping review to examine the geriatric HIV care literature for older adults living with HIV. A scoping review methodology is appropriate due to the broad nature of co-design and allows exploration of knowledge across study designs [22, 23]. The modified Arksey and O’Malley [23, 24] scoping review frameworks and Joanna Briggs Institute (JBI) Manual for scoping review studies framework [25] will be followed. The proposed framework includes the following 1) developing a research question, 2) developing a search strategy, 3) evidence screening and study selection, 4) data extraction, 5) data analysis; and 6) consultation [23–25]. The review reporting will follow the Preferred Reporting Items for Systematic Reviews and Meta-analysis for Protocols (PRISMA-P) [26] and the PRISMA extension for scoping reviews (PRISMA-ScR) [27] guidelines. The preliminary literature search will begin in April 2024, and we anticipate this review will be completed in late 2024.

### Stage 1: Developing a research question

The research team consisting of researchers and a patient partner were consulted to develop and clarify the research questions. This scoping review aims to understand various co-design and community-based research methods implemented within the geriatric HIV care literature to inform future methodologies in geriatric HIV research and co-design studies. This scoping review outlined here will address the following research questions:

1. What is the extent, range, and nature of geriatric-HIV research methods that have used co-design methods?
2. What co-design methods have been used to develop HIV interventions for individuals aged 50 or older?
3. Who is being excluded from existing co-design studies for older adults living with HIV (as defined by the PROGRESS+ characteristics)?

### Stage 2: Developing a search strategy

Literature will be found using a search strategy created and drafted by an Information Specialist and Health Science Librarian (TBD) in consultation with the review team. The following text words and subject headings will be included in the search, as they pertain to concepts addressed by the research questions: ‘older adults’ ‘HIV’ ‘co-design’ ‘co-creation’ ‘co-production’ ‘community-based research’ and ‘geriatric-HIV interventions’. To ensure a breadth of understanding, we will define older adults in the context of HIV care as aged 50 years or older [28]. The search will be limited to articles written or translated to English. Papers published prior to the 2003 will not be considered within the search to find the most up-to-date literature informing future research, due to resource constraints. To minimize search errors and enhance comprehensiveness, the search will be peer-reviewed using the Peer Review of Electronic Search Strategies [29]. After the search strategy is refined and finalized, the search will be conducted by the librarian in MEDLINE(R) ALL (in Ovid, including Epub Ahead of Print, In-Process & Other Non-Indexed Citations, Ovid MEDLINE(R) Daily) and then translated it into NLM’s PubMed OVID Embase + Embase Classic, EBSCO’s CINAHL Complete, Clarivate’s Web of Science Core Collection, and Elsevier’s Scopus.

Relevant gray literature will be found by handsearching using similar search terms as the scientific search through Google Scholar, Open Grey, open Google searches and relevant websites, such as WHO, UK National Research Register, CADTH’s “Grey Matters”, New York Academy of Medicine’s Grey Literature Report, the Canadian Medical Association InfoBase and the National Institute for Heath and Care Excellence–Guidance.

Due to the broad nature of this search, included studies’ reference lists will also be searched to identify any missed studies in the search. Forward citation searching, consisting of a citation index that cites eligible studies in the scoping review [30], will also be conducted to identify any studies missed in the search.

### Stage 3: Evidence screening and study selection

Endnote will be used to remove duplicate articles and Covidence will be used to complete screening of articles [31, 32]. Two reviewers will independently screen and review articles (PB and KMK), first completing title/abstract screening (level 1-screening) and then full-text article screening (level 2-screening). Any discrepancies in screening will be discussed by the reviewers and resolved by team-based discussion. Articles will be included and excluded based on criteria listed in Table 1.

**Table 1:** Inclusion and exclusion criteria.

Criteria	Inclusion	Exclusion
Population:	Eligible studies will include individuals aged 50 or older living with HIV.	Populations other than older adults living with HIV.
	In order to be included, >1 older adult with HIV will need to have been involved in co-design as a participant.	Older adults living with HIV who were not involved in co-design methods and a participant in the study.
Concept:	Studies that describe co-design or community-based research in the context of models of care for older adults living with HIV.	Studies that focus on models of care not developed in community partnership or co-design with older adults living with HIV.
		Studies that focus on a co-design or community-based research that have not yet been implemented.
		Studies that report on co-design methods in HIV care not specific to older adults.
Context:	Studies that identify and/or employ strategies for co-design methods to develop HIV interventions for individuals aged 50 or older.	Protocol papers or papers refining or developing conceptual models, methods and frameworks to guide co-design strategies with older adults with HIV will be excluded.
Study designs:	All study designs using empirical data collection (i.e.., qualitative, quantitative or mixed method methodologies) will be eligible for inclusion, except for case reports.	Non-empirical literature and relevant grey literature (e.g., conference abstracts, theses and dissertations).
		We will search for the full-text articles of conference abstracts and study protocols that fulfil our eligibility criteria during a hand-search.
Time periods:	Studies completed in the past 20 years will be included.	Studies completed prior to 20 years ago (before 2003) will be excluded.
Setting:	Studies in any healthcare setting or country will be considered for inclusion.	
Language:	Only full-text papers written in English will be considered for inclusion.	Literature not available in full-text in English.

### Stage 4: Data extraction

Data extraction will be completed by two independent reviewers using a data extraction form in Covidence. Similar to screening, the data extraction will be an iterative process, with final categories decided upon as reviewers become more familiar with data and studies reviewed [24]. Data extracted will include, but is not limited to, author last name, year, study type, setting, country, methods, methodology, characteristics of intervention, delivery method (i.e., virtual, in-person, telephone), participant characteristics, provider characteristics, results, and key conclusions. First, each reviewer will extract data from a random sample of five included studies. Once >75% agreement is reached between the two reviewers, then half of the included studies will be screened. Reviewers will then meet to discuss discrepancies and if poor agreement is found, the data abstraction form will be clarified, and conflicts will be resolved by a third reviewer. Following this, the senior responsible author will check all data extraction to ensure agreement. Appraisal for risk of bias and quality of the studies will not be performed, as per the Joanna Briggs Institute Manual [22, 33].

### Stage 5: Data analysis

Data extracted will be analyzed using a content analysis as recommended for scoping reviews [34] Explicit rules of coding will be used to create content categories. The data will be coded manually by the research team and will be grouped based on the main components of the studies extracted including main components of the co-design model, methodology, framework used, population, location of study, population age, associated methods (eg, one-on-one interviews, workshops, etc.), and other PROGRESS-Plus characteristics (eg, race, ethnicity, language, culture, level of education) [20].

### Stage 6: Consultation

The findings of this scoping review are intended to guide co-design with older persons living with HIV. As such, through co-design, we will consult with several knowledge users including community partners and diverse individuals with lived experience with HIV aged 50 years or older. Prior to data extraction, we will invite key stakeholders, including HIV care specialists, advisory committee members including older adults living with HIV, and HIV community organizations (i.e., healthcare organizations, social service agencies, housing support and shelters) to share feedback on the data extraction table to ensure the data adequately reflects the community-based perspective. Our advisory committee consists of individuals involved in geriatric HIV policymaking, research, advocacy, and individuals with lived experience with HIV and aging. Consulting community partners will provide insights beyond the literature reviewed to gain deeper understanding on methodology and methods used and potential areas for improvement with co-design methods. Going forward, insights on co-design methodology from community partners will be used to guide meaningful co-design of solutions to HIV care for older adults.

### Limitations

Only papers published or translated to English will be included in the study search criteria, thus articles not published in English or translated to English that are relevant to co-design may not be included. Co-design methods have been defined in various terms by different research, including but not limited to, co-production and co-creation, [35] and thus articles using different terminology regarding co-design, such as co-build, or human-centred design, may be excluded from the search.

## Discussion

Our upcoming scoping review focuses on synthesizing the use of co-design methods for research involving older adults living with HIV. Co-design methods empower older individuals living with HIV to actively participate in shaping their own care experiences, thereby enhancing patient-centered care [36–38]. The objectives, methods, and data extraction process are outlined to systematically explore the extent, range, and nature of geriatric HIV research that has employed methods with co-design approaches, and will identify who may be excluded from these studies, keeping in mind PROGRESS-Plus characteristics [20]. The importance of consultation with key stakeholders, including community partners and individuals with lived experience, is emphasized to ensure that the insights gained from the review are translated into meaningful co-design of virtual solutions for HIV care for older adults. This review will result in a comprehensive synthesis of the existing literature, offering researchers and healthcare professionals a deeper understanding of how co-design methods have been used to address the unique needs of older adults living with HIV. By identifying gaps in current research and highlighting successful approaches to co-design with older adults with HIV, this review can inform the development of evidence-based strategies for improving the quality of care and support for this older adult population. Successful approaches will emphasize principles from the SPOR Patient Engagement consultation [21] leading to meaningful engagement in co-design research. Additionally, the review’s focus on inclusivity and cultural sensitivity underscores its potential to promote more equitable and person-centered healthcare, addressing disparities faced by older adults living with HIV from diverse backgrounds. Ultimately, this scoping review serves as a crucial step in shaping the future of geriatric HIV care, where approaches to co-design can play a pivotal role in enhancing healthcare delivery, to improve overall well-being.

## Supporting information

S1 Checklist PRISMA-P (Preferred Reporting Items for Systematic review and Meta-Analysis Protocols) 2015 checklist: Recommended items to address in a systematic review protocol*. (PDF)
